# Progressive substitution of posttransplant cyclophosphamide with bendamustine: A phase I study in haploidentical bone marrow transplantation

**DOI:** 10.1002/jha2.20

**Published:** 2020-05-26

**Authors:** Emmanuel Katsanis, Keri Maher, Denise J. Roe, Richard J. Simpson

**Affiliations:** ^1^ Department of Pediatrics University of Arizona Tucson Arizona; ^2^ Department of Immunobiology University of Arizona Tucson Arizona; ^3^ Department of Medicine University of Arizona Tucson Arizona; ^4^ Department of Pathology University of Arizona Tucson Arizona; ^5^ The University of Arizona Cancer Center Tucson Arizona; ^6^ Banner University Medical Center Tucson Arizona; ^7^ Department of Epidemiology and Biostatistics University of Arizona Tucson Arizona; ^8^ Department of Nutritional Sciences University of Arizona Tucson Arizona

**Keywords:** haploidentical BMT, myeloablative, posttransplant bendamustine

## Abstract

We have initiated a single center phase I study in patients with hematologic malignancies progressively substituting day +4 posttransplant cyclophosphamide (PT‐CY) with bendamustine (PT‐BEN) following myeloablative conditioning (MAC) and T‐cell replete haploidentical bone marrow transplantation (haplo‐BMT). We report herein our interim analysis of our first three cohorts PT‐CY (mg/kg)/PT‐BEN (mg/m^2^): 40/20, 20/60, and 0/90. All patients have tolerated PT‐CY/BEN well with no dose limiting toxicities. Compared to contemporaneous controls undergoing haplo‐BMT with the same MAC regimens but only PT‐CY, we have observed earlier trilineage engraftment (*P = *.002 neutrophils, *P = *.014 platelets) and a lower incidence of cytomegalovirus reactivation (*P = *.016) in the PT‐CY/BEN cohorts. After substituting day +4 PT‐CY with PT‐BEN, the registered trial (www.clinicaltrials.gov; NCT02996773) is proceeding to replace day +3 PT‐CY with PT‐BEN with a view to identifying further evidence on the potential advantages of PT‐BEN.

## 1 INTRODUCTION

Posttransplant cyclophosphamide (PT‐CY) has been met with enthusiasm in the haploidentical bone marrow transplantation (haplo‐BMT) arena as it reduces rates of graft‐versus‐host disease (GvHD). However, of two‐thirds of patients with an absence of acute GvHD (aGvHD) after nonmyeloablative conditioned haplo‐BMT, half relapsed [1]. Moreover, cytomegalovirus (CMV) reactivation and BK cystitis remain noteworthy complications following PT‐CY [2]. We have previously demonstrated in an experimental murine haplo‐BMT model that posttransplant bendamustine (PT‐BEN) was equally protective against early GvHD and had advantages in late GvHD and in graft‐versus‐leukemia (GvL) effects when compared to PT‐CY [3]. We further documented that PT‐BEN mitigated GvHD even in the absence of T regulatory cells. PT‐BEN was associated with an increased myeloid compartment in vivo and with enhanced suppressive function of myeloid‐derived suppressor cells while impairing the proliferation of T and B cells. Our results advocated for the consideration of PT‐BEN as a therapeutic platform for clinical implementation in haplo‐BMT. We therefore initiated a phase I trial to evaluate PT‐BEN in haplo‐BMT by progressively substituting PT‐CY with PT‐BEN and report herein the hitherto findings from our first three cohorts.

## 2 PATIENTS AND METHODS

### 2.1 Patients

Patients were treated as part of an institutional review board (IRB)–approved phase I/Ib single‐institution trial using a standard 3+3 dose escalation design with six‐dose level cohorts (Table 1). The first three cohorts, reported here, consisted of a combination of progressively reduced doses of CY and increased doses of BEN on day +4 post‐BMT with the day +3 dose of CY remain unchanged. Eligible patients were between 8 and 60 years, who had no matched‐related donor and no readily available matched‐unrelated donor, met organ criteria allowing for myeloablative conditioning, and had no evidence of active untreated infection. Patients undergoing haplo‐BMT and fulfilling eligibility criteria for the study, but unwilling to receive PT‐BEN, were asked to participate as PT‐CY controls for the clinical endpoints. Moreover, in order to proceed from one phase I cohort to the next, there is a 28‐day period of observation. Therefore, patients requiring haplo‐BMT during those intervals were also enrolled as PT‐CY controls upon consent. All haplo‐BMTs were performed at Banner University Medical Center in Tucson, Arizona between January 2017 and October 2019. Ten patients were transplanted on the pediatric service (ages 9.2‐24.7 years) and seven on the adult service (ages 26.1‐44.6 years).

**TABLE 1:** Phase I/Ib trial of PT‐bendamustine

	Day +3		Day +4	
Cohort #	CY mg/kg	BEN, mg/m^2^	CY, mg/kg	BEN, mg/m^2^
1	50	0	40	20
2	50	0	20	60
3	50	0	0	90
4	40	20	0	90
5	20	60	0	90
6	0	90	0	90
Phase Ib	0	90	0	90

All acute lymphoblastic leukemia (ALL) patients had negative bone marrow minimal residual disease assessment by flow cytometry prior to initiation of conditioning. Two of the patients with acute myeloid leukemia and two with undifferentiated and bispecific leukemia were in morphologic remission. Two patients with chronic myelogenous leukemia in chronic phase had failed tyrosine kinase inhibitor therapy, while one was in remission following induction chemotherapy for lymphoid blast crisis. One patient in cohort #1 had refractory non‐Hodgkin lymphoma (NHL‐anaplastic large cell lymphoma) and was in partial remission at the time of transplant.

### 2.2 Transplant procedure

All patients received a MAC regimen. According to diagnosis and disease characteristics, we decided either for fractionated total body irradiation (TBI) followed by fludarabine (FLU) or for busulfan (BU), fludarabine (FLU), and melphalan (MEL) combination (Table 2) [4]. Nine patients were conditioned with fractionated TBI of 200 cGy BID given on days −8, −7, and −6 (1200 cGy total dose with lungs shielded to 900 cGy by custom cerrobend blocking), followed by FLU 30 mg/m^2^ on days −5, −4, −3, and −2 [4, 5]. Eight patients received BU at 0.8 mg/kg IV every 6 hours for a total of 12 doses (days −8 to −6), targeting an average area under the curve of 1000–1100 µMol/min for the duration of the course. BU was followed by FLU 30 mg/m^2^ on days −5, −4, −3, and −2 and MEL 100 mg/m^2^ on day −2 [4, 6].

**TABLE 2:** Patient, disease, and transplant characteristics

	PT‐CY/BEN (n = 9)	PT‐CY (n = 8)	*P*
Age, median year (range)	21.4 (9.2‐42.9)	30.7 (16.8‐44.6)	.11
Male gender, n (%)	5 (55.6)	6 (75)	.62
Race/ethnicity, n (%)			.99
White Hispanic	5 (55.5)	4 (50)	
Native American	0	0	
African American	1 (11.1)	2 (25)	
White	3 (33.3)	2 (25)	
Diagnosis, n (%)			.58
B‐ALL	4 (55.6)	3 (37.5)	
T‐ALL	1 (11.1)	1 (12.5)	
AML	1 (11.1)	1 (12.5)	
Bi/Un‐AL	1 (11.1)	1 (12.5)	
CML	1 (11.1)	2 (25)	
NHL	1 (11.1)	0 (0)	
Pretransplant status, n (%)			.99
CR1	3 (33.3)	2 (25)	
CR2	2 (22.2)	3 (37.5)	
>CR2	2 (22.2)	1 (12.5)	
Other	2 (22.2)	2 (25)	
Disease risk index, n (%)			.99
Low	1 (11.1)	1 (12.5)	
Intermediate	8 (88.9)	7 (87.5)	
High	0	0	
Lansky/Karnofsky, median (range)	90 (60‐100)	90 (50‐100)	.99
HCT comorbidity index, median (range)	0 (0‐4)	1.5 (0‐4)	.11
Conditioning, n (%)			.99
TBI‐FLU	5 (55.5)	4 (50)	
BU‐FLU‐MEL	4 (44.4)	4 (50)	
BMT service			.64
Pediatric	6 (66.7)	4 (50)	
Adult	3 (33.3)	4 (50)	
Graft composition median (range)			.07
CD34+ × 10^6^/kg	4.05 (2.1‐7.4)	2.70 (1.5‐4.8)	
RBC incompatibility, n (%)			.64
None	4 (44.4)	5 (62.5)	
Major	4 (44.4)	3 (37.5)	
Minor	1 (11.1)	0 (0)	
Donor age, median year, (range)	27 (17.9‐57.6)	37.7 (20.9‐62.4)	.74
Donors of male recipients, n (%)			.18
Mother	1 (20)	2 (33.3)	
Father	2 (40)	0 (0)	
Sister	1 (20)	1 (16.6)	
Brother	0	3 (50)^#^	
Son	1 (20)	0	
Donors of female recipients, n (%)			.47
Mother	2 (50)	0	
Father	0	1 (50)	
Sister	1 (25)	0	
Brother	1 (25)	1 (50)	
Donors match, n (%)			.13
5/10	8 (88.9)	4 (50)	
6/10	0	3 (37.5)	
7/10	1 (11.1)	1 (12.5)	

Abbreviations: ALL, acute lymphoblastic leukemia; AML, acute myeloid leukemia; BEN, bendamustine; Bi/U, bispecific or undifferentiated acute leukemia; BU, busulfan; CML, chronic myeloid leukemia; CR, complete remission; CY, cyclophosphamide; FLU, fludarabine; MEL, melphalan; MMF, mycophenolate mofetil; NHL, non‐Hodgkin lymphoma; Tacro, Tacrolimus; TBI, total body irradiation.

# It includes one half‐sibling.

### 2.3 GvHD prophylaxis

All patients received T‐replete bone marrow followed by PT‐CY 50 mg/kg on day +3. On day +4, cohort #1 patients received 40 mg/kg PT‐CY immediately followed by PT‐BEN 20 mg/m^2^, cohort #2 patients were treated with PT‐CY 20 mg/kg followed by PT‐BEN 60 mg/m^2^, and cohort #3 patients received only PT‐BEN 90 mg/m^2^ (Table 1). The eight patients in the control group received the standard PT‐CY 50 mg/kg on days +3 and +4. Additionally, all patients were given mycophenolate mofetil on day +5 through day +28 and tacrolimus starting on day +5. In the absence of GvHD, tacrolimus was decreased starting day +70 to +90 and discontinued by day +120 to +180. GvHD was graded according to the consensus criteria for grading acute and chronic GvHD [7, 8].

### 2.4 Supportive care

Antifungal prophylaxis was administered in all patients. Patients received IV pentamidine for pneumocystis jirovecii and acyclovir for herpes simplex and varicella virus prophylaxis. No CMV prophylaxis with letermovir or other agents was used. Bi‐weekly polymerase chain reaction monitoring for CMV and weekly for adenovirus, Epstein‐Barr virus (EBV), and human herpes virus‐6 (HHV‐6) were performed until discharge from hospital and subsequently at least every other week during the first 100 days following transplant. All patients were transplanted in positive pressure HEPA‐filtered rooms on a HEPA‐filtered unit and encouraged to walk laps on the unit daily.

### 2.5 Donor selection

Donors were first degree relatives who were HLA‐haploidentical based on high‐resolution typing at HLA‐A, ‐B, ‐Cw, ‐DRB1, and ‐DQB1 (Table 2). None of the patients had antidonor HLA antibodies. There were seven major ABO incompatibilities that required donor RBC reduction using Hespan® (6% hetastarch in 0.9% sodium chloride injection) for RBC sedimentation and one minor incompatibility requiring plasma reduction [9]. ABO incompatibilities were comparable between groups.

### 2.6 Engraftment and donor chimerism monitoring

Granulocyte‐colony stimulating factor (G‐CSF) was started on day +5 at 5 µg/kg/day until an absolute neutrophil count (ANC) of 2.5 × 10^9^/L was achieved for three consecutive days. Day of myeloid engraftment was defined as the first of three consecutive days with an ANC of ≥ 0.5 × 10^9^/L. Day of platelet engraftment was considered the first of three consecutive days with platelet counts of ≥ 20 × 10^9^/L with no platelet transfusions administered in the previous 7 days. Donor chimerism was evaluated on days +28, +100, +180, and +365 by short tandem repeats on peripheral blood or bone marrow.

### 2.7 Statistical analysis

Comparisons of patient characteristics and outcome variables between the PT‐CY and PT‐CY/BEN groups were performed using Fisher's exact tests for categorical variables and Mann‐Whitney/Wilcoxon rank sum tests for continuous variables. Time to event endpoints were estimated using cumulative incidence curves and Kaplan‐Meier curves, with comparisons using log‐rank tests. The association between the number of CD34^+^ × 10^6^ cells infused/kg with time to neutrophil or platelet engraftment was estimated using linear regression analysis.

## 3 RESULTS

### 3.1 Patient, disease, and transplant characteristics

Patient characteristics are summarized in Table 2. Baseline characteristics such age, gender, race/ethnicity, diagnosis, pretransplant remission status, donor age, disease risk index or performance status, and HCT comorbidity index were comparable between groups (*P = *n.s.). Ethnic and/or racial minorities constituted 70.6% of all patients, the majority of whom were Hispanic. ALL was the most frequent diagnosis (52.9%). Seven of the nine (77.8%) ALL patients were conditioned with TBI‐FLU, while six of the eight (75%) patients with other diagnoses received BU‐FLU‐MEL (Table 2).

### 3.2 Engraftment and chimerism

**FIGURE 1:**
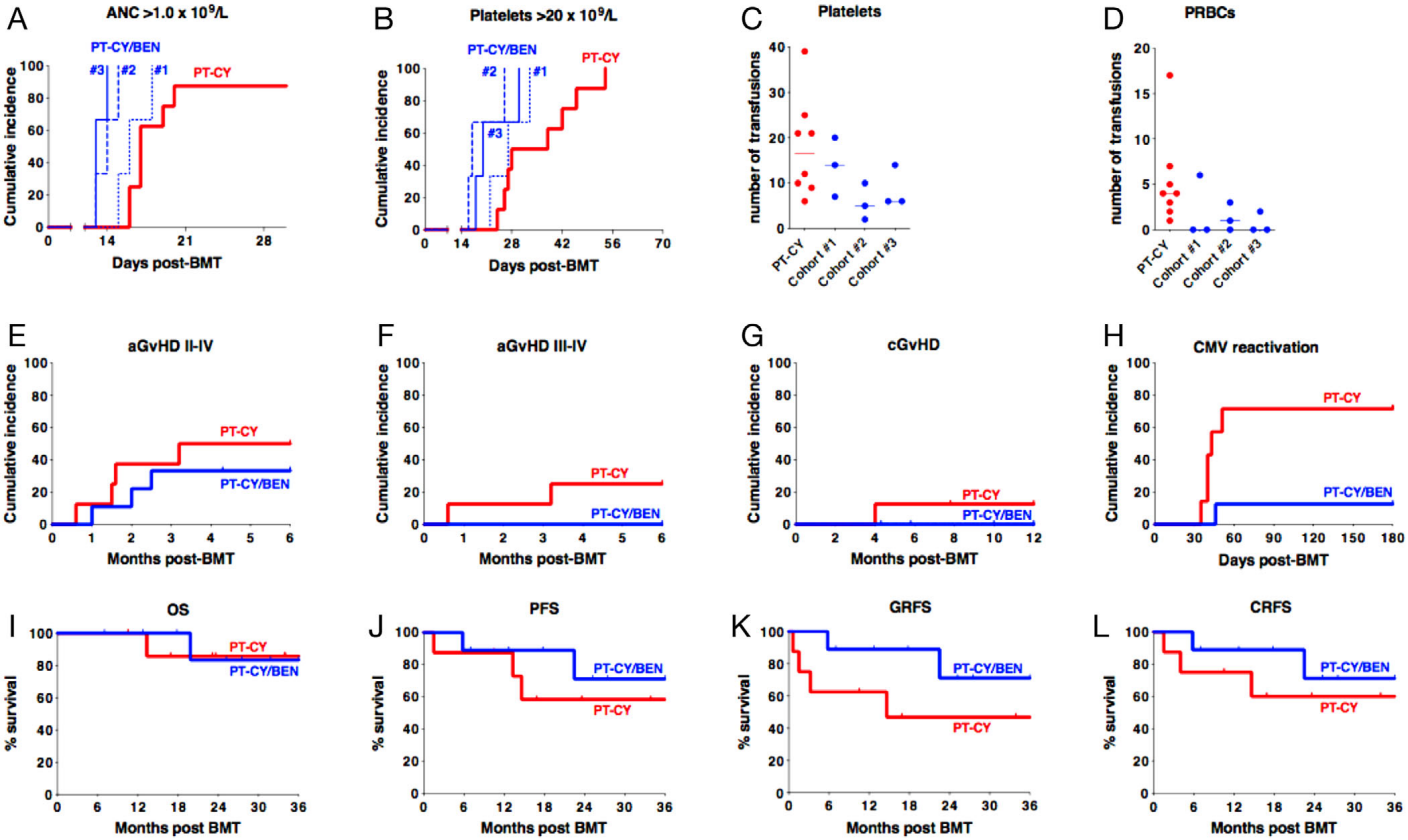
A, Time to an absolute neutrophil count (ANC) of 1 × 10^9^/L. Median time PT‐CY, 17 days; Cohort #1, 16 days (*P = *.14); Cohort #2, 14 days (*P = *.0004); Cohort 3, 13 days, (*P = *.0005). B, Time to a platelet count of 20 × 10^9^/L. Median time PT‐CY, 33 days; Cohort #1, 27 days (*P = *.18); Cohort #2, 17 days (*P = *.0007); Cohort #3, 20 days (*P = *0.07). C, Number of units of platelets transfused. Median PT‐CY, 16.5 units; Cohort #1, 14 units, (*P* = n.s.); Cohort #2, 5 units (*P = *.05); Cohort #3, 6 units, (*P = *n.s.). D, Number of units of packed red blood cells (PRBCs) transfused. Median PT‐CY, 4 units; Cohort #1, 0 units (*P* = n.s.); Cohort #2, 1 unit (*P = *.07); Cohort #3, 0 units (*P = *.03). E, Cumulative incidence of grades II‐IV acute graft‐versus‐host disease (aGvHD); (*P = *n.s.). F, Cumulative incidence of grades III‐IV aGvHD; (*P = *n.s.). G, Cumulative incidence of chronic GvHD; (*P = *n.s.). H, Cumulative incidence of CMV viremia; (*P = *.016). I, Overall survival (OS); (*P =* n.s.) J, Progression‐free survival (PFS); (*P = *n.s.) K, Graft‐versus‐host‐free‐relapse‐free survival (GRFS); (*P = *n.s.). L, Chronic graft‐versus‐host‐free‐relapse‐free survival (CRFS); (*P = *n.s.).

PT‐CY/BEN patients received a median of 4.05 × 10^6^/kg CD34^+^ cells compared to 2.7 × 10^6^/kg in the PT‐CY group (*P = *.07) (Table 2). All patients that received PT‐CY/BEN had early trilineage engraftment, while one patient in the PT‐CY group failed to engraft despite receiving an adequate number of CD34^+^ cells (4.8 × 10^6^/kg) and required a second transplant. Interestingly, no correlation was observed between the number of CD34^+^cells/kg infused and time to neutrophil or platelet engraftment (Figure S1). The median time to an ANC of 1.0 × 10^9^/L was achieved progressively earlier in each PT‐CY/BEN cohort even though the median number of CD34^+^cells/kg infused was comparable between cohorts (4.6, 3.7, and 4.05 × 10^6^/kg, *P = *n.s.) (Figure 1A). Similarly, the latter PT‐CY/BEN cohorts demonstrated earlier platelet engraftment (Figure 1B). Consequently, PT‐CY/BEN patients required fewer platelet and red blood cell transfusions (Figure 1C,D). All PT‐CY/BEN patients showed complete donor chimerism in their day +28 bone marrows and in peripheral blood on days +100 and +180 as did all patients that have reached their 1‐year follow‐up.

### 3.3 Graft versus host disease

The cumulative incidence of grade II‐IV aGvHD was 33.3% following PT‐CY/BEN (66.7%, 33.3%, and 0% by cohort) compared to 50% in controls (Figure 1E). No grade III‐IV aGvHD was seen in PT‐CY/BEN compared to 25% in controls (Figure 1F and Table S1). While none of the PT‐CY/BEN patients developed lower gastrointestinal (GI) aGvHD of any stage, four had skin and one had upper GI involvement. Moreover, no patients receiving PT‐CY/BEN developed signs of chronic GvHD (cGvHD), while one PT‐CY control patient developed extensive cGvHD (Figure 1G).

### 3.4 Transplant‐related toxicities

None of the patients that received PT‐CY/BEN developed major transplant‐related complications in the early post‐BMT period, such as sinusoidal obstruction syndrome, transplant‐associated thrombotic microangiopathy (TA‐TMA) or idiopathic pneumonia syndrome, and none required admission to the intensive care unit (ICU), continuous renal replacement therapy (CRRT), vasopressors, or mechanical ventilation (Table S2). In contrast, two PT‐CY control patients were admitted to ICU with one requiring mechanical ventilation. Additionally, two control patients developed end‐stage renal disease necessitating CRRT followed by chronic renal dialysis, one from BK viral nephritis and cidofovir adverse effect and the other from BK and TA‐TMA.

### 3.5 Infections

There were five Gram‐positive and no Gram‐negative bacteremias in four patients receiving PT‐CY/BEN seen between day +5 and day +100 compared to three Gram‐positive and one Gram‐negative in four PT‐CY patients, all of which responded to appropriate antibiotic therapy. All the Gram‐positive bacteremias occurred before day +35 (median day +12). There were no documented fungal infections in either group (Table S3). CMV reactivation was significantly less common in trial patients receiving PT‐CY/BEN with only one out of eight at risk (seropositive recipient and/or seropositive donor) reactivating CMV (Table 3), compared to 71.4% of at‐risk PT‐CY patients (Figure 1H). The majority of CMV reactivations peaked between day +40 and +50 (Figure S2A) with viral loads of between 10^3^ and 10^5^ IU/mL (Figure S2B). All patients responded to antiviral therapy (Figure S2C). BK viruria was documented in four (50%) PT‐CY patients having > 5 × 10^8^ viral copies/mL and symptoms of BK hemorrhagic cystitis, compared to two PT‐CY/BEN patients (22.2%) both from cohort #1 (with the highest PT‐CY dose) (*P = n.s*.) (Figure S2D). The two PT‐CY patients that developed end‐stage renal failure requiring dialysis also had significant BK viremia of 6000 and 7500 viral copies. None of the patients had clinically significant reactivation of EBV, HHV‐6, or adenovirus warranting therapeutic intervention.

**TABLE 3:** CMV reactivation following haplo‐BMT

	PT‐CY/BEN	PT‐CY	*P*
CMV status recipient/donor, n (%)			
R+/D+	1/4 (25)	3/4(75)	.49
R+/D−	0/4 (0)	2/2 (100)	.06
R−/D+	0/0 (0)	0/1 (50)	.99
Total/at risk	1/8 (12.5)	5/7 (71.4)	.04
R−/D−	0/1(0)	0/1 (0)	*–*

### 3.6 Survival, nonrelapse mortality, and relapse

With a median follow‐up of 25.2 months (range 7‐36.7) in the PT‐CY/BEN group and 23.3 months (10.5‐39.2) in the PT‐CY group, the overall survival at 2 years is similar at 83.3% for PT‐CY/BEN trial patients compared to 85.7% for the PT‐CY control group (*P = *n.s.) (Figure 1I). Progression‐free survival at 2 years is also comparable with 71.1% for PT‐CY/BEN group versus 58.3% for those receiving PT‐CY (*P = *n.s.) (Figure 1J). There was no nonrelapse mortality in the PT‐CY/BEN group, while one patient in the PT‐CY group died of chronic GvHD and multiorgan failure on day +404. Two patients in each group have relapsed resulting in similar probabilities of relapse at two years of 28.9% for PT‐CY/BEN versus 30% for PT‐CY (*P = *n.s.). The probability of GvHD‐free‐relapse‐free survival and chronic GvHD‐free‐relapse‐free survival at 2 years for PT‐CY/BEN patients is 71.1% and 71.1%, compared to 46.9% and 66.7% for controls (*P = *n.s.) (Figure 1K,L).

## 4 DISCUSSION

Our preclinical murine study in haplo‐BMT demonstrated that PT‐BEN compared to PT‐CY promoted earlier and higher neutrophil counts, similar protection against early GvHD, superior control of late GvHD, and enhanced GvL effects [3]. Building on these preclinical findings, we initiated a phase I clinical trial to progressively substitute PT‐CY with PT‐BEN following haplo‐BMT. PT‐CY has been considered safe against hematopoietic stem cells as these cells express high levels of aldehyde dehydrogenase, thus detoxifying CY [10, 11]. However, BEN has multiple activities as it contains a mechlorethamine group, butyric acid side chain, and a benzimidazole ring. The alkylating properties provided by the mechlorethamine group are similar to CY, while the butyric acid increases BEN's water solubility and the benzimidazole ring is believed to function as a purine analogue, affording antimetabolic characteristics [12, 13, 14]. We were therefore cautious in designing the phase I trial, only affording gradual progression in de‐escalation of CY and escalation of BEN starting with day +4, while leaving the dose of CY on day +3 unchanged. Our initial experience has indicated that not only did PT‐CY/BEN not hamper donor engraftment, but actually facilitated it. In fact, with each cohort, as the dose of PT‐BEN was increased and PT‐CY decreased, we observed earlier neutrophil engraftments. PT‐CY/BEN patients demonstrated a trend of improvement of aGVHD in each cohort (Table S1) with no patients developing grade III‐IV aGvHD or cGvHD (Figure 1F,G). Therefore, our preliminary clinical experience mirrors our preclinical studies in mice.

Viral infections contribute to substantial transplant‐related morbidity and mortality in patients undergoing haplo‐BMT, with CMV being the leading culprit [15, 16, 17]. We observed a significant decrease in CMV reactivation in our trial patients compared to those receiving PT‐CY alone (Figure 1H). As was seen with our control patients, the incidence of CMV reactivation in recipients of haplo‐BMT with PT‐CY is particularly high with as many as two‐thirds of patients at risk (serological positive recipients and/or donors) developing CMV viremia and CMV disease reported to occur in one‐third of these patients [2, 16, 17]. As expected, given the increased risk of hemorrhagic cystitis with exposure to CY, we observed a trend toward lower BK viruria with only two patients, both from cohort #1 that received 90% of the PT‐CY dose, developing BK viruria compared to half of those treated with PT‐CY (Figure S2D). Two of the control patients also had BK viremia, which may have contributed to their renal failure [18]. Therefore, our results corroborate that BK is a significant complication of PT‐CY reported to occur in one‐third to one‐half of patients undergoing haplo‐BMT with PT‐CY [19]. If PT‐BEN emerges as a safe alternative to PT‐CY, it may have the added advantage of reduced hemorrhagic cystitis and renal complications associated with BK viremia.

The use of PT‐CY originated from studies in murine models performed at Johns Hopkins University in the early 2000s [20]. Our laboratory has pioneered the use of PT‐BEN, first in an experimental murine haplo‐BMT model [3], and has now translated this approach clinically. Although our findings are preliminary and limited, our interim analysis provides encouraging evidence that PT‐BEN may emerge as a viable alternative to PT‐CY. With the trial proceeding to replacement of day +3 PT‐CY with PT‐BEN, we will hopefully be able to provide further evidence of the potential advantages of PT‐BEN.

## AUTHOR CONTRIBUTIONS

Emmanuel Katsanis designed the research and clinical trial and is the PI. He analyzed and reviewed the data and wrote the manuscript. Keri Maher is a co‐PI of the clinical trial and edited the manuscript. Denise J. Roe is a co‐PI of the trial and performed the statistical analysis of the data. Richard J. Simpson is a co‐PI of the clinical trial, advised on the study, and edited the manuscript.

## CONFLICT OF INTEREST

There are no conflicts of interest, financial or otherwise, involving any of the authors regarding the submission or publication of this manuscript.

## Supporting information

Supporting Information
