# Supplementary material for: Progressive substitution of posttransplant cyclophosphamide with bendamustine: A phase I study in haploidentical bone marrow transplantation
Source: EJHaem. 2020 May 26;1(1):286–92. doi: 10.1002/jha2.20 (PMC9176108; doi:10.1002/jha2.20)
Supplement: Supplementary file 1 — Supporting Information [file JHA2-1-286-s001.docx]

**Supporting Information**

**Supplemental Tables**

**Table SI. Acute GVHD grading**

|  | 0 | I | II | III | IV |
| --- | --- | --- | --- | --- | --- |
| Cohort #1 n=3 | 1 | 0 | 2 | 0 | 0 |
| Cohort #2 n=3 | 1 | 1 | 1 | 0 | 0 |
| Cohort #3 n=3 | 2 | 1 | 0 | 0 | 0 |
|  |  |  |  |  |  |
| PT-CY n=8 | 1 | 3 | 2 | 1 | 1 |

**Table SII. Transplant related toxicity**

| First 100 days post-BMT n (%) | ICU | MV | CRRT/RD | TMA | SOS | TRM |
| --- | --- | --- | --- | --- | --- | --- |
| Cohort #1 n=3 | 0 | 0 | 0 | 0 | 0 | 0 |
| Cohort #2 n=3 | 0 | 0 | 0 | 0 | 0 | 0 |
| Cohort #3 n=3 | 0 | 0 | 0 | 0 | 0 | 0 |
|  |  |  |  |  |  |  |
| PT-CY n=8 | 2 (25) | 1 (12.5) | 2 (25) | 1 12.5) | 0 | 0 |

BMT, bone marrow transplant; ICU, intensive care unit; MV, mechanical ventilation; CRRT/RD, continuous renal replacement therapy/renal dialysis; TMA, thrombotic microangiopathy; SOS, sinusoidal obstruction syndrome; TRM, transplant related mortality

**Table SIII. Bacteremias and fungal infections**

|  | PT-CY/BEN | PT-CY | *P* |
| --- | --- | --- | --- |
|  | **n=9** | **n=8** |  |
| Gram positive bacteremia n (%) | **5 (55.6)** | **3 (37.5)** | *0.64* |
| Staphylococcus epidermidis | 1 | 1 |  |
| Staphylococcus hominis |  | 1 |  |
| Streptococcus mitis | 2 |  |  |
| Streptococcus sanguinis | 1* |  |  |
| Rothia dentocariosa | 1* |  |  |
| Enterococcus gallinarum |  | 1 |  |
| Gram negative bacteremia | **0** | **1 (12.5)** | *0.47* |
| Klebsiella pneumoniae | 0 | 1 |  |
| Fungal | **0** | **0** | *0.99* |

*, both bacteria cultured the same day from the same patient

**Supplemental Figures**


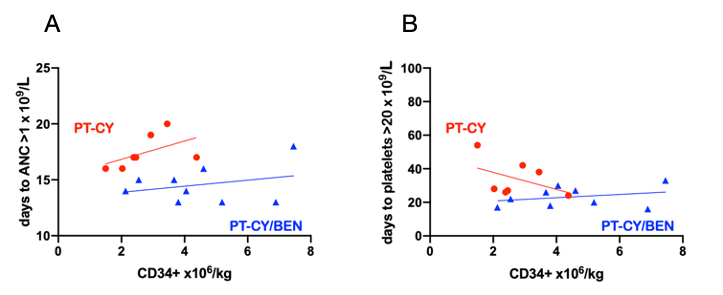


**Figure S1. Lack of association between the number of CD34^+^ cells infused/kg and time to neutrophil or platelet engraftment. (A) Time to ANC of 1.0 x 10^9^/L.** Linear regression analysis. PT-BEN/CY R2=0.08, p=0.45; PT-CY R2=0.27 P=0.23. **(B) Time to platelet count of 20 x 10^9^/L.**  PT-BEN/CY R2=0.08, P=0.45; PT-CY R2=0.19 P=0.32.


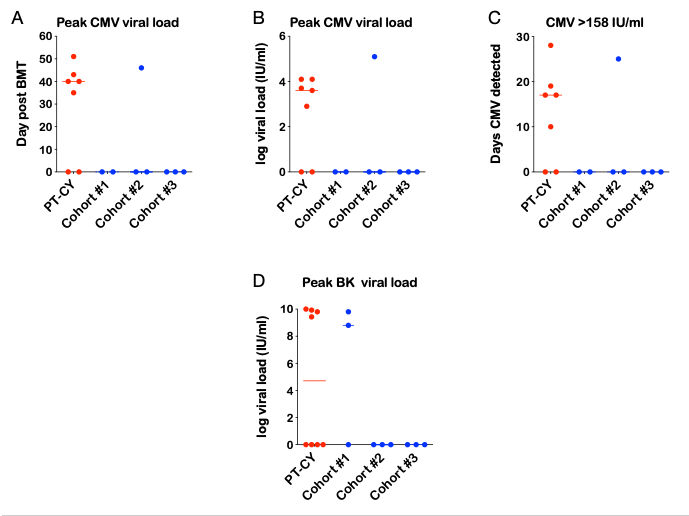


**Figure S2. CMV viremia and BK viruria.** **(A) Day post-BMT of peak CMV blood viral load. (B) Log of peak CMV blood viral load detected. (C) Number of days CMV viremia was detectable. (D) Log of peak urine BK viral load detected.**
